# Smart ICUs: The Role of Artificial Intelligence in Modern Intensive Care Units

**DOI:** 10.7759/cureus.106934

**Published:** 2026-04-13

**Authors:** Nikita Singh

**Affiliations:** 1 Critical Care Medicine, Fortis Escorts Heart Institute, New Delhi, IND

**Keywords:** artificial intelligence, deep learning, intensive care unit, machine learning, outcome

## Abstract

Artificial intelligence (AI) is increasingly transforming intensive care medicine by enabling advanced analysis of complex clinical data generated in intensive care units (ICUs). This review explores current and emerging applications of AI in ICU practice, including sepsis prediction, mechanical ventilation management, acute kidney injury (AKI) forecasting, haemodynamic monitoring, and prognostication. AI-based models have demonstrated the ability to improve early detection of complications, support clinical decision-making, and optimise resource utilisation. However, challenges such as limited interpretability, data integration constraints, and the need for prospective validation continue to hinder widespread clinical adoption. A comprehensive narrative review was conducted using publications from January 2015 to June 2025. Combinations of the terms "artificial intelligence", "machine learning", "deep learning", "intensive care unit", "critical care", "clinical decision support", and "sepsis prediction" were used to search PubMed, Scopus, and Google Scholar. Peer-reviewed original research, systematic reviews, and meta-analyses reporting on the practical uses or clinical validation of AI tools in ICUs were given precedence, while studies focusing solely on algorithm development without clinical integration were excluded. Sepsis, mechanical ventilation, AKI, haemodynamic monitoring, and prognostication are among the thematic areas of application that organise the review. AI has shown significant utility across ICU domains, including early prediction of complications, forecasting mechanical ventilation duration, risk stratification, haemodynamic instability alerts, and mortality prognostication. Models trained on real-world ICU datasets have demonstrated high predictive accuracy and potential for early intervention. However, challenges such as model interpretability, data fragmentation, and ethical concerns remain.

## Introduction and background

With the advent of computers, Alan Turing was among the first to propose the concept of machines simulating human intelligence. He introduced the "Turing Test" to evaluate whether machines could think and analyse in a manner comparable to humans. Later, John McCarthy defined artificial intelligence (AI) as "the science and engineering of making intelligent machines." These foundational concepts have evolved over time, leading to the development of several key subfields, including machine learning (ML), deep learning (DL), natural language processing (NLP), and computer vision (CV) [1].

NLP and CV enable computers to extract and interpret human language data and visual information, facilitating automated analysis and supporting clinical decision-making processes [2].

ML, as a subset of AI, enables systems to learn from data, identify patterns, and improve performance over time through computational algorithms. It utilises a wide range of data inputs to generate predictive outputs, allowing for autonomous decision-making and clinical recommendations. With continuous cycles of data input, output generation, and iterative learning, these models can predict outcomes with increasing accuracy. Deep neural networks represent a more advanced form of ML, using multiple processing layers to analyse complex data structures. This enhanced capability allows for improved pattern recognition and model development without explicit programming, forming a specialised subset known as DL [3].

Supervised learning models are widely used for outcome prediction and risk stratification, whereas unsupervised learning enables pattern recognition in high-dimensional datasets. DL models, particularly neural networks, have demonstrated exceptional capability in processing time-series physiological data and imaging modalities, making them especially relevant in intensive care settings.

The growing complexity of intensive care medicine has led to an unprecedented volume of patient data generated through continuous monitoring systems, laboratory investigations, and electronic health records. Clinicians are often required to make rapid decisions based on large and dynamic datasets, which may exceed the limits of traditional clinical interpretation. AI offers the potential to integrate and analyse these multimodal data streams, identify clinically relevant patterns, and support early detection of complications while optimising treatment strategies.

**Figure 1:**
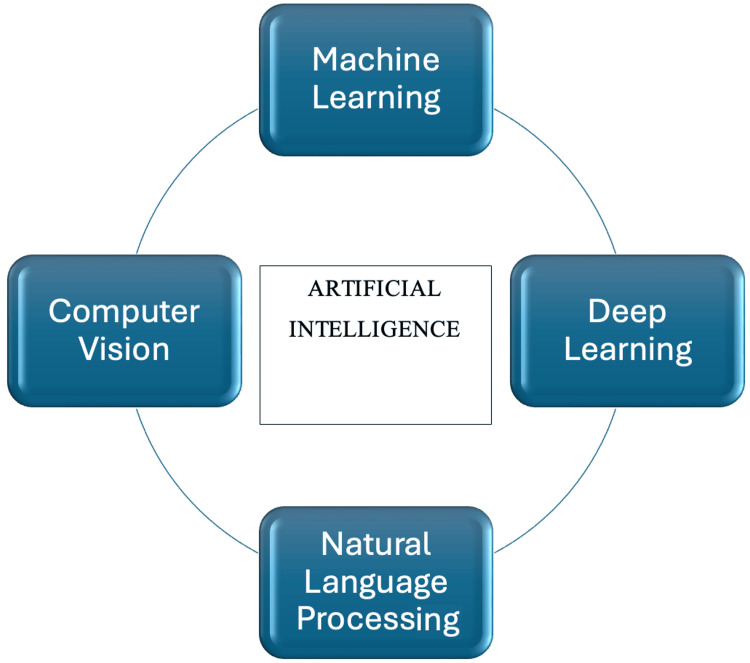
Components of artificial intelligence Images were created by the author using Microsoft Word (Microsoft® Corp., Redmond, WA).

Therefore, understanding the current applications, capabilities, and limitations of AI in the intensive care unit is increasingly important for modern critical care practice. The core components of AI are illustrated in Figure 1.

## Review

Application of AI in ICUs

AI in Sepsis

Goh et al. have used AI to develop the Sepsis Early Risk Assessment (SERA) algorithm, which utilises structured data and unstructured clinical notes and data to assess sepsis risk. SERA consists of an interlinked diagnostic algorithm that determines the probability of sepsis and an early prediction algorithm that determines the risk of developing sepsis in the subsequent 4, 6, 12, 24, and 48 hours. They had utilised the hospital's electronic medical records (EMR) system. In the EMR, an NLP algorithm was applied to classify the data into seven categories. These data were used to determine whether a patient will develop sepsis within the next 4, 6, 12, 24, and 48 hours. They also compared this algorithm with other predictive scoring systems, such as systemic inflammatory response syndrome (SIRS), sequential organ failure assessment (SOFA), quick SOFA (qSOFA), and modified early warning system (MEWS), to predict sepsis and sepsis-related mortality. In a retrospective validation of ICU data, the model demonstrated high predictive accuracy, particularly 12 hours before sepsis onset [3].

Shashikumar et al. used the Deep Artificial Intelligence Sepsis Expert (DeepAISE) for early sepsis prediction. This model automatically learned the predictive features of various data and clinical interactions to make an early prediction of sepsis. It can accurately predict the likelihood of sepsis in ICU patients up to 12 hours in advance [4].

These models utilise time-series physiological data and clinical variables to identify subtle temporal patterns preceding sepsis onset, enabling earlier prediction compared to conventional scoring systems.

AI models can be developed to aid in early prediction and recognition of conditions such as ventilator-associated pneumonia and central line-associated bloodstream infections. They will aid healthcare workers in monitoring, planning, and initiating timely interventions [5].

A 2025 study published in JMIR Medical Informatics developed a DL-based system using convolutional neural networks (CNNs) to predict the onset of sepsis from multivariate time-series ICU data. Drawing from a large retrospective dataset of over 36,000 ICU admissions, the model incorporated continuous monitoring parameters to capture complex patterns associated with early sepsis. The CNN model demonstrated excellent performance, predicting sepsis up to six hours before the traditional clinical diagnosis. When benchmarked against established tools such as the SOFA and qSOFA scores, the AI model not only outperformed them in predictive accuracy but also offered earlier identification of high-risk patients. These findings reinforced the potential of explainable DL systems to support real-time sepsis alerts in the ICU [6].

**Figure 2:**
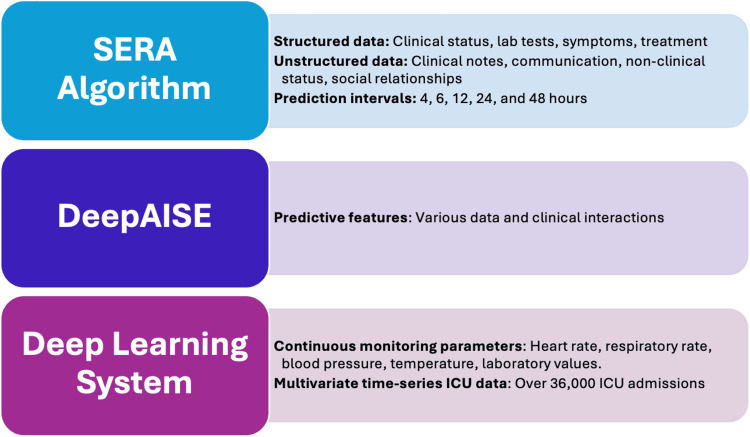
Key variables used in each AI model for sepsis prediction AI: artificial intelligence; DeepAISE: Deep Artificial Intelligence Sepsis Expert; SERA: Sepsis Early Risk Assessment

Key variables used in each AI model for sepsis prediction are illustrated in Figure 2.

AI in Mechanical Ventilation

With recent advances in AI, it will play a crucial role in the mechanical ventilation of critically ill patients. It can help determine various ventilatory parameters to optimise the patient's ventilation, prevent ventilator-associated adverse effects, and assess the likelihood of a successful weaning trial. The newer closed-loop ventilation mode could address complex issues, such as preventing ventilator-induced lung injury by continuously adapting to lung mechanics and the patient's condition while assessing weaning success and extubation readiness [7].

Mendiratta et al. had analysed two AI models: the regression model and the binary classification model. A regression model was developed to predict ventilation duration in days. In contrast, a binary classification model was developed to divide patients into groups of short-term (< 3 days) or long-term (> 3 days) mechanical ventilation. A three-day cutoff was considered, as it demonstrated clinical significance and a higher risk of developing ventilator-associated pneumonia. They concluded that proposed AI models, equipped with robust solutions for predicting ventilator days, may also aid clinical decision-making and resource allocation [8].

Wang et al. used an AI model to predict mechanical ventilation duration in patients with acute respiratory distress syndrome (ARDS) in the ICU. They utilised data from the Medical Information Mart for Intensive Care (MIMIC-IV) and the AmsterdamUMCdb to develop a prediction model based on seven supervised ML algorithms. Ultimately, they employed three descriptive ML explanation methods. Seven ML algorithms were used. Compared to all the models, the extreme gradient boosting (XGB) model had the most accurate prediction performance. They concluded that ML models can help manage patients on mechanical ventilation in the ICU [9].

These ML models analyse dynamic respiratory parameters and patient-specific variables to predict ventilation duration and guide personalised ventilatory strategies.

A study published in Frontiers in Medicine developed a two-stage AI model to predict the optimal timing for weaning patients from mechanical ventilation. The model utilised a combination of clinical variables, respiratory parameters, and spontaneous breathing trial data to support decision-making across different phases of the weaning process. By incorporating dynamic patient data and stage-specific prediction algorithms, the system accounted for the evolving physiological status of critically ill patients. Using large ICU datasets, the model demonstrated strong predictive performance, with area under the curve (AUC) values exceeding 0.84 across both stages. Importantly, when implemented through a clinical decision-support dashboard, the AI-assisted system showed good agreement with clinician judgment while improving efficiency. Its use was associated with a reduction in mechanical ventilation duration by approximately 21 hours, suggesting a meaningful impact on patient outcomes and ICU resource utilisation. These findings highlight the potential of AI to standardise and optimise ventilator weaning strategies by providing objective, data-driven guidance. In clinical practice, variability in clinician experience and decision-making can influence weaning outcomes; AI-based tools may help reduce this variability and support more consistent care. Furthermore, earlier and appropriate weaning can reduce the risk of ventilator-associated complications, shorten ICU stay, and improve overall patient recovery. Such systems may be particularly valuable in high-demand settings where efficient resource utilisation and timely decision-making are critical [10].

AI in Acute Kidney Injury (AKI)

AKI is a prevalent and critical complication seen in ICU patients, with high morbidity, mortality, and resource utilisation, especially when continuous renal replacement therapy (CRRT) is required.

The study by Tan et al. demonstrates the growing potential of AI in ICU triage, particularly in forecasting critical events, such as AKI and the subsequent need for CRRT. The use of long short-term memory networks for time series data and BioMedBERT for clinical text enables more nuanced and temporally aware risk predictions. Achieving high predictive accuracy with a lead time of over 12 hours underscored the model's clinical relevance. Furthermore, interpretability through Shapley Additive exPlanations (SHAP) analysis enhances its transparency and clinical acceptability by revealing key contributing features, such as urine output, anion gap, and phosphate. Importantly, the ability to identify patients at high risk of renal deterioration early may allow for timely, individualised interventions. This study reinforces the promise of multimodal AI models in real-time ICU decision-making. It supports their integration into clinical decision support systems (CDSS) for stratifying AKI risk [11].

Fragasso et al. developed a random forest model using 36 EMR-based features to predict AKI more than 48 hours after paediatric cardiac surgery. The model demonstrated high accuracy with key predictors reflecting both renal and surgical stressors. They concluded that, while the model demonstrates strong performance, its single-centre design and absence of external validation restrict its broader applicability [12].

A study published in Frontiers in Cardiovascular Medicine developed multiple ML models to predict the risk of AKI in patients with acute myocardial infarction using large-scale ICU datasets from the MIMIC-III and MIMIC-IV databases. The study evaluated several algorithms, including random forests, support vector machines, and XGB, and incorporated a wide range of clinical variables, including laboratory values, comorbidities, and physiological parameters. A total of 3,882 patients were analysed, of whom approximately 28% developed AKI during hospitalisation. Among the models tested, the random forest algorithm demonstrated the best predictive performance, achieving strong discrimination in both internal and external validation cohorts. Feature importance analysis using SHAP identified key predictors, including serum creatinine, blood glucose, platelet count, and atrial fibrillation, highlighting the roles of both renal and systemic factors in AKI development. This study underscores the potential of ML to enhance early risk stratification of AKI in critically ill patients by integrating multidimensional clinical data. By enabling early identification of high-risk patients, such models may facilitate timely interventions, personalised treatment strategies, and improved clinical outcomes. Furthermore, the use of externally validated datasets strengthens the reliability and generalisability of these AI-driven approaches in real-world ICU settings [13].

AI in Haemodynamic Monitoring

Hatib et al. developed an ML algorithm using arterial pressure waveform data to predict intraoperative hypotension (MAP < 65 mmHg) up to 15 minutes before onset. They trained on over 500,000 minutes of waveform data and validated in a separate cohort. By analysing over 3,000 features per cardiac cycle, the algorithm detected subtle haemodynamic changes preceding hypotension, enabling early identification of circulatory instability. This tool, later commercialised as the Hypotension Prediction Index, represents one of the first real-time AI applications in anaesthesiology, supporting proactive haemodynamic management during surgery [14].

The model analyses high-frequency arterial waveform data to detect early haemodynamic instability before clinically apparent hypotension occurs.

O'Driscoll et al. evaluated the prognostic value of AI-derived echocardiographic parameters in patients presenting with chest pain. Using the Ultromics EchoGo Core 2.0 platform, the study automatically measured left ventricular ejection fraction (LVEF) and global longitudinal strain (GLS) from resting transthoracic echocardiograms in 296 patients. Over a median follow-up of 7.8 years, both LVEF and GLS were strong, independent predictors of major adverse cardiac events and mortality. This study highlights the utility of AI-enabled echocardiography for long-term risk prediction, supporting its integration into routine patient assessment for suspected cardiac pathology [15].

De Raat et al. conducted a prospective study to evaluate the performance of a fully automated, tablet-based monoplane echocardiographic tool (AutoEF) designed for rapid, point-of-care quantification of LVEF and stroke volume (SV). The study included 35 adult patients undergoing routine transthoracic echocardiography. AutoEF's performance was benchmarked against a reference computer-based system (Tomtec), using both monoplane (apical four-chamber) and biplane (apical four- and two-chamber) measurements. Results demonstrated a strong correlation between LVEF estimation and both monoplane and biplane analysis. However, the correlation between SV was more modest. With this, the authors concluded that AutoEF offered a promising solution for fast, user-independent LVEF measurement in bedside settings, particularly in resource-limited environments or during acute care scenarios [16].

**Figure 3:**
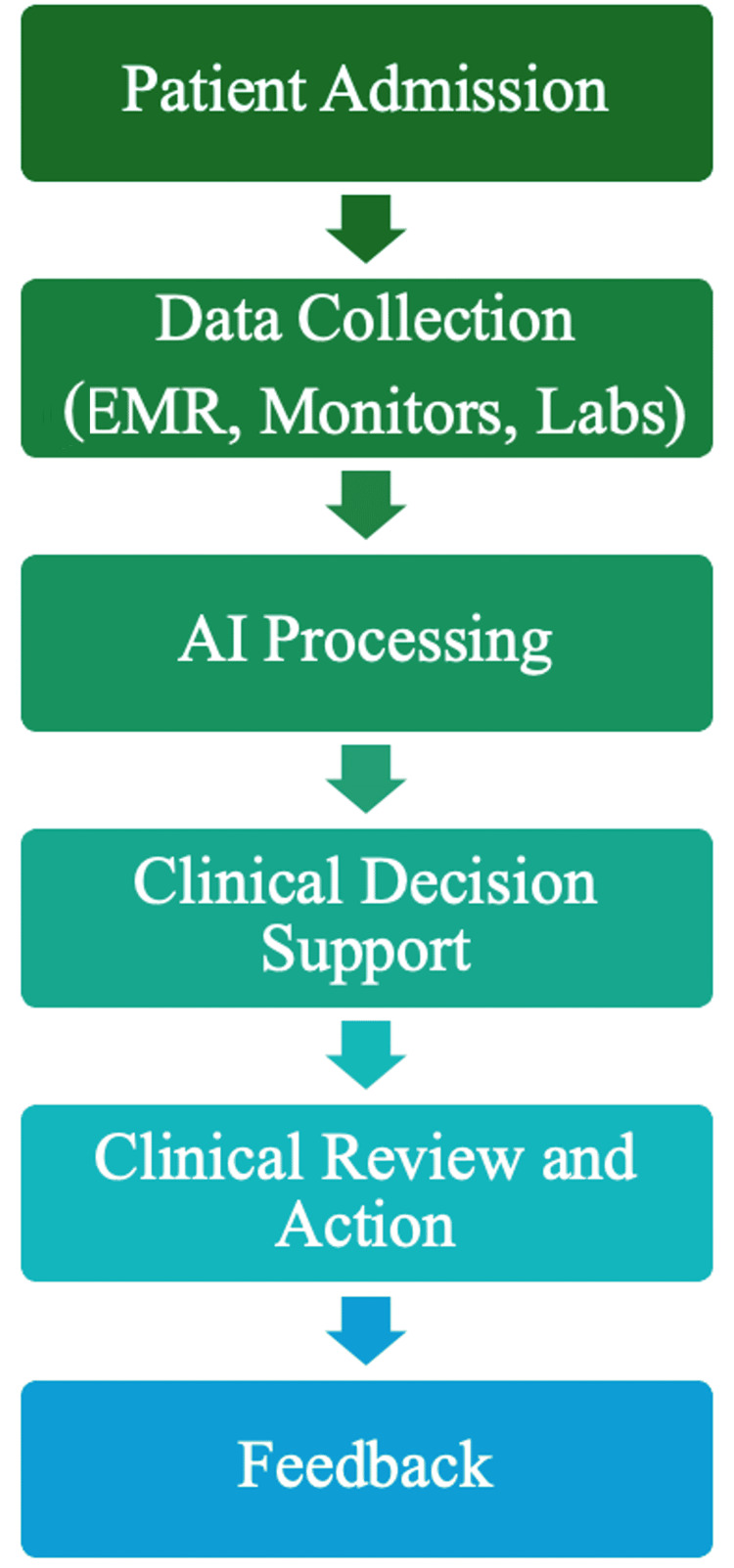
Flowchart illustrating the stages of AI integration in intensive care units, from data collection through AI processing to clinical decision-making and feedback AI: artificial intelligence; EMR: electronic medical records

The process of AI integration in ICU practice is demonstrated in Figure 3.

AI in Prognostication and Outcome

As outlined in a narrative review by Medical Informatics (2022), AI-driven models are being developed to predict critical outcomes of scenarios such as sepsis, AKI, and ARDS, with performance often exceeding that of traditional scoring systems. CDS tools powered by ML assist intensivists in the early detection of patient deterioration and optimising resource allocation, particularly in high-acuity or resource-constrained settings. In parallel, AI-based image analysis, such as DL algorithms for chest X-rays and point-of-care ultrasound, enables faster, more consistent interpretation, thereby reducing interobserver variability. NLP tools are also being used to extract structured clinical insights from unstructured EMR notes, providing comprehensive timelines of patient data for informed decision-making. The review emphasises the growing importance of explainable AI in improving clinician trust and model interpretability, as well as the need for multicenter validation and interdisciplinary collaboration among clinicians, data scientists, and engineers [17].

Awad et al. introduced an ensemble ML framework, Ensemble Machine Learning Model for Mortality Prediction Inside Intensive Care Unit (EMPICU), to predict in-hospital mortality in ICU patients within the first six hours of admission. Using data from 11,722 adult ICU patients in the MIMIC-II database, the study evaluated multiple algorithms against traditional severity scoring systems. The EMPICU‑RF model significantly outperformed standard scores, achieving superior discrimination early in the patient's ICU stay. The authors demonstrated that, despite missing data from the initial hours, sufficient physiologic and laboratory signals exist within six hours to enable effective mortality prediction [18].

Calvert et al. developed a predictive algorithm, AutoTriage, to identify patients at high risk of death in the medical ICU using EMR data. The model was constructed using a minimal set of eight routinely collected clinical variables. Trained on the MIMIC-III database, the model aimed to predict mortality within a 12-hour window and demonstrated strong performance with both sensitivity and specificity around 80%. Compared to established scoring systems, AutoTriage provided superior discrimination, particularly for short-term mortality prediction, making it a valuable tool for early ICU risk stratification and potential intervention planning. The key strength of the AutoTriage model is its simplicity and interpretability. The use of limited but high-yield variables enabled real-time applicability without the need for complex imaging or extensive lab panels. Each variable was weighted and scored independently, allowing clinicians to understand individual contributions to risk and better tailor bedside decision-making. Furthermore, the algorithm's reliance on data available within routine workflows facilitates its integration into existing ICU infrastructure. This study demonstrates how targeted ML applications utilising EMR data can significantly enhance early ICU prognostication with the potential to inform triage and resource allocation strategies [19].

These models utilise routinely collected clinical variables to generate early risk scores, enabling timely intervention and improved ICU triage.

Discussion

The integration of AI in critical care has opened new horizons for clinicians to understand, monitor, and respond to the dynamic clinical conditions of critically ill patients. From early prediction of sepsis and AKI to real-time assessment of haemodynamic status and prognostication of outcomes, AI has demonstrated the potential to enhance both the timeliness and precision of ICU interventions [3-5,13].

The key applications of AI in the ICU have been summarised in Table 1, and the AI integration workflow is illustrated in Figure 3.

**Table 1:** Summary of key applications of AI in ICUs EMR: electronic medical records; AKI: acute kidney injury; CRRT: continuous renal replacement therapy; ICU: intensive care unit; XGB: extreme gradient boosting; LSTM: long short-term memory

Domain	Clinical Application	AI Model/Approach	Data Source	Key Clinical Benefit	References
Sepsis Prediction	Early diagnosis and risk stratification	SERA, DeepAISE, CNN-based deep learning models	EMR, clinical notes, time-series ICU data	Early prediction up to 6-12 hours before onset and improved clinical decision support	[3,4,6]
Mechanical Ventilation	Duration prediction and weaning optimisation	Regression models, classification models, XGB, two-stage AI weaning model	MIMIC-IV, AmsterdamUMCdb, ICU datasets	Accurate prediction of ventilation duration and optimised weaning timing, reducing ventilation duration	[8-10]
Acute Kidney Injury	AKI prediction and CRRT requirement forecasting	LSTM, BioMedBERT, Random Forest, ML risk prediction models	Time-series data, EMR, MIMIC-III/IV databases	Early risk stratification, improved prediction of AKI, and timely intervention	[11-13]
Haemodynamic Monitoring	Prediction of hypotension and circulatory instability	Hypotension Prediction Index, ML-based echocardiographic tools	Arterial waveform data, echocardiography	Early detection of haemodynamic instability and improved peri-operative management	[14-16]
Prognostication	ICU mortality prediction and risk stratification	EMPICU, AutoTriage models	EMR variables	Early identification of high-risk patients and improved ICU triage	[18,19]

Recent advances in mechanical ventilation further highlight the clinical applicability of AI. ML models have demonstrated the ability to predict ventilation duration and optimise ventilatory strategies using large ICU datasets [8,9].

Additionally, more sophisticated approaches, such as the two-stage AI weaning model, have shown strong predictive performance and have been associated with a reduction in mechanical ventilation duration while maintaining agreement with clinician judgement [10].

These findings suggest that AI can support more objective and consistent ventilatory management, potentially reducing complications associated with prolonged ventilation.

Similarly, in the context of AKI, AI models integrating time-series physiological data and EMRs have demonstrated strong predictive capabilities [11].

More recent ML approaches using large databases, such as MIMIC-III and MIMIC-IV, have further improved risk stratification by incorporating multidimensional clinical variables and enabling external validation [12]. These models also utilise explainability techniques, such as SHAP analysis, which enhance transparency by identifying key contributing features. Such developments are particularly important in improving clinician trust and facilitating real-world implementation of AI systems.

However, the road to widespread implementation remains complex. Although many models show promising results, they are often developed using retrospective datasets and may be prone to overfitting, requiring further validation across diverse populations and healthcare settings. Variability in data quality, patient demographics, and clinical practices can significantly influence model performance. Clinicians also require a deeper understanding of these algorithms to appropriately interpret and apply their outputs in clinical practice. Furthermore, translating AI models into real-world clinical settings remains challenging due to issues such as a lack of external validation, data heterogeneity, and integration difficulties into existing healthcare systems. Integration into ICU workflows is another major challenge, often complicated by fragmented data systems and limited interoperability with existing EMRs. Ethical concerns, including algorithmic bias, accountability, and patient data privacy, further complicate adoption. Addressing these issues is essential to ensure safe and equitable deployment of AI in critical care [17-20].

Despite these barriers, the future of AI in critical care remains promising. The next generation of AI tools is likely to be more clinician-friendly and patient-adaptive, enabling dynamic responses to evolving clinical conditions. Greater emphasis on explainable AI, prospective validation, and interdisciplinary collaboration between clinicians, data scientists, and engineers will be essential. With thoughtful implementation and continued innovation, AI has the potential to become an integral component of modern intensive care, supporting clinicians in delivering safer, more efficient, and personalised care.

Prospective validation and integration into real-time clinical workflows remain key areas for future research.

## Conclusions

AI is steadily emerging as a powerful tool in intensive care medicine, where rapid and accurate decision-making is critical. Across a wide range of applications, including early prediction of complications (e.g., sepsis and AKI), real-time haemodynamic monitoring, and automated interpretation of complex clinical data, AI has demonstrated significant potential to enhance clinical performance and improve patient outcomes. However, despite these advancements, translation into routine clinical practice remains limited due to challenges such as a lack of model transparency, variability in data quality, integration difficulties with existing healthcare systems, and the need for rigorous prospective validation. Addressing these limitations through improved transparency and stronger collaboration between clinicians and data scientists will be essential for the successful implementation of AI in modern critical care.
